# Lipid Emulsion Attenuates Acetylcholine-Induced Relaxation in Isolated Rat Aorta

**DOI:** 10.1155/2015/871545

**Published:** 2015-07-27

**Authors:** Seong-Ho Ok, Soo Hee Lee, Jongsun Yu, Jungchul Park, Il-Woo Shin, Youngju Lee, Hyunhoo Cho, Mun-Jeoung Choi, Jiseok Baik, Jeong-Min Hong, Jeong Yeol Han, Heon Keun Lee, Young-Kyun Chung, Ju-Tae Sohn

**Affiliations:** ^1^Department of Anesthesiology and Pain Medicine, Gyeongsang National University Hospital, Gyeongsang National University School of Medicine, Jinju 660-715, Republic of Korea; ^2^Department of Anesthesiology and Pain Medicine, Gyeongsang National University Hospital, Jinju 660-702, Republic of Korea; ^3^Department of Oral and Maxillofacial Surgery, Gyeongsang National University Hospital, Jinju 660-702, Republic of Korea; ^4^Department of Anesthesiology and Pain Medicine, Pusan National University Hospital, Biomed Research Institute, Pusan National University School of Medicine, Pusan 602-739, Republic of Korea; ^5^Institute of Health Sciences, Gyeongsang National University School of Medicine, Jinju 660-715, Republic of Korea

## Abstract

We investigated the effect of Lipofundin MCT/LCT and Intralipid on acetylcholine-induced nitric oxide- (NO-) mediated relaxation in rat aorta to determine which lipid emulsion (LE) is more potent in terms of inhibition of NO-induced relaxation. Dose-response curves of responses induced by acetylcholine, the calcium ionophore A23187, and sodium nitroprusside were generated using isolated rat aorta with or without LE. The effect of Lipofundin MCT/LCT on acetylcholine-induced endothelial nitric oxide synthase (eNOS) phosphorylation in human umbilical vein endothelial cells (HUVECs) was investigated using western blotting. Lipofundin MCT/LCT (0.1 and 0.2%) attenuated acetylcholine-induced relaxation in endothelium-intact aorta with or without tiron, whereas 0.2% Intralipid only inhibited relaxation. Lipofundin MCT/LCT inhibited relaxation induced by the calcium ionophore A23187 and sodium nitroprusside in endothelium-intact aorta, but Lipofundin MCT/LCT had no effect on sodium nitroprusside-induced relaxation in the endothelium-denuded aorta. Combined pretreatment with l-arginine plus Lipofundin MCT/LCT increased acetylcholine-induced maximal relaxation in endothelium-intact aorta compared with Lipofundin MCT/LCT alone. l-Arginine attenuated Lipofundin MCT/LCT-mediated inhibition of acetylcholine-induced eNOS phosphorylation in HUVECs. Taken together, Lipofundin MCT/LCT attenuated acetylcholine-induced NO-mediated relaxation via an inhibitory effect on the endothelium including eNOS, which is proximal to activation of guanylyl cyclase.

## 1. Introduction

Intravenous lipid emulsion (LE) has been used for parenteral nutrition when the oral and enteral route is not available. LEs such as either Intralipid 20% containing 100% long-chain triglycerides or Lipofundin MCT/LCT 20% containing 50% long-chain triglycerides and 50% medium-chain triglycerides are effective for treatment of cardiovascular collapse induced by a toxic dose of local anesthetics including bupivacaine, levobupivacaine, ropivacaine, mepivacaine, and lidocaine [1–7]. In addition, LE is also used as a nonspecific antidote to treat systemic toxicity of other drugs that otherwise lack a specific antidote [7].

Triacylglycerol, fatty acids, and triglycerides decrease nitric oxide (NO) release [8–11]. LE containing only long-chain triglycerides has no effect on blood pressure, whereas LE containing both medium-chain triglycerides and long-chain triglycerides causes an increase in blood pressure [12]. Intralipid increases blood pressure and inhibits acetylcholine-induced NO-mediated vasorelaxation [13–17]. In addition, intravenous administration of LE produces increased left ventricular systolic pressure, and pretreatment with the nitric oxide synthase (NOS) inhibitor N^w^-nitro-l-arginine-methyl ester (L-NAME) abolishes the LE-induced left ventricular systolic pressure increase, suggesting that LE-induced increases in left ventricular systolic pressure may be due to inhibition of NO release [18]. Recently, we reported that LE-mediated reversal of a toxic dose of levobupivacaine-induced vasodilation in isolated rat aorta appears to be partially associated with attenuation of endothelial NO release induced by levobupivacaine, suggesting that LE-mediated reversal of a toxic dose of levobupivacaine-induced NO-mediated vascular collapse may partially contribute to recovery of vascular tone from vascular collapse caused by systemic toxicity of levobupivacaine [19, 20]. As local anesthetics induce NO release that may contribute to vascular collapse at a toxic dose of the anesthetic, the magnitude of LE-mediated inhibition of NO-induced relaxation may be considered an important factor in the recovery of vascular tone following a toxic dose of local anesthetics that induces vascular collapse [19–22]. However, whether the magnitude of LE-mediated attenuation of acetylcholine-induced NO-mediated relaxation in isolated vessels is associated with the components (long-chain triglycerides and medium-chain triglycerides) of LEs such as Intralipid 20% and Lipofundin MCT/LCT 20% is unknown. In other words, the type of LE (Intralipid 20% and Lipofundin MCT/LCT 20%) that is more potent for attenuating acetylcholine-induced NO-mediated relaxation remains unknown. Thus, we examined the effect of Intralipid and Lipofundin MCT/LCT on acetylcholine-induced NO-mediated relaxation of isolated rat aorta to determine whether LE-mediated attenuation of NO-induced relaxation is dependent on the fatty acid component (long-chain and medium-chain fatty acids) of LE. We also elucidated the associated cellular mechanism. Based on previous reports, we tested the hypothesis that Lipofundin MCT/LCT attenuates acetylcholine-induced NO-mediated relaxation more than Intralipid [12, 18].

## 2. Materials and Methods

All experimental procedures and protocols were approved by the Institutional Animal Care and Use Committee at Gyeongsang National University. All experimental procedures were performed in accordance with the Guide for the Care and Use of Laboratory Animals prepared by the National Academy of Sciences.

### 2.1. Preparation of Aortic Rings for Tension Measurement

Preparation of aortic rings for tension measurement was performed as described previously [23]. Male Sprague-Dawley rats weighing 250–300 g were anesthetized with intramuscular injection of Zoletil 50 (10 mg/kg). The descending thoracic aorta was dissected free, and surrounding connective tissues and fat were removed under a microscope in a Krebs solution bath (118 mM NaCl, 4.7 mM KCl, 1.2 mM MgSO_4_, 1.2 mM KH_2_PO_4_, 2.4 mM CaCl_2_, 25 mM NaHCO_3_, and 11 mM glucose). The aorta was cut into 2.5 mm rings, and the endothelium was removed from some aortic rings by inserting a 25-gauge needle tip into the lumen of the rings and gently rubbing the ring for a few seconds. The cut aortic rings were suspended on Grass isometric transducers (FT-03, Grass Instrument, Quincy, MA, USA) under a 3.0 g resting tension in a 10 mL Krebs bath at 37°C and aerated continuously with 95% O_2_ and 5% CO_2_ to maintain the pH within the range of 7.35–7.45. The rings were equilibrated for 120 min, and the bathing solution was changed every 40 min. As soon as phenylephrine (10^−7^ M)-induced contraction had stabilized, the endothelial integrity was confirmed by the observation of >70% relaxation with the addition of acetylcholine (10^−5^ M). Only one concentration-response curve elicited by endothelium-dependent relaxing agents (acetylcholine and the calcium ionophore A23187) and an endothelium-independent relaxing agent (sodium nitroprusside) was created from each ring in all experiments. The contractile response induced by isotonic 60 mM KCl was measured for some aortic rings and used as a reference value for phenylephrine-induced contraction.

### 2.2. Experimental Protocol

The experimental protocol was designed to investigate the effect of LE (Intralipid and Lipofundin MCT/LCT) alone and combined treatment with l-arginine plus Lipofundin MCT/LCT on the vasodilation induced by endothelium-dependent vasodilators (acetylcholine and the calcium ionophore A23187) and an endothelium-independent vasodilator (sodium nitroprusside) in isolated rat aorta. Lipid emulsion alone (Intralipid or Lipofundin MCT/LCT: 0.1 or 0.2%) or l-arginine (10^−4^ M) plus Lipofundin MCT/LCT (0.2%) was directly added to the organ bath 20 min before phenylephrine-induced contraction. Subsequently, the endothelium-intact and endothelium-denuded rings were precontracted with 3 × 10^−7^ and 10^−7^ M phenylephrine, respectively. When the contraction induced by phenylephrine (3 × 10^−7^ M) had stabilized in the endothelium-intact aorta, incremental concentrations of acetylcholine (3 × 10^−8^ to 10^−5^ M), the calcium ionophore A23187 (10^−9^ to 10^−6^ M), and sodium nitroprusside (3 × 10^−10^ to 10^−7^ M) were directly added to the organ bath to generate the concentration-response curves in the presence or absence of LE (Intralipid or Lipofundin MCT/LCT: 0.1 or 0.2%) alone and l-arginine (10^−4^ M) plus Lipofundin MCT/LCT (0.2%). After the calcium ionophore A23187 (10^−7^ M) had produced maximal relaxation in the control condition, we did not add higher concentrations (3 × 10^−7^ and 10^−6^ M) of A23187 to the organ bath because high concentrations produce vasoconstriction in control conditions [24]. When the contraction to phenylephrine (10^−7^ M) had stabilized in the endothelium-denuded aorta, incremental concentrations of sodium nitroprusside (10^−10^ to 10^−7^ M) were directly added to the organ bath to generate the concentration-response curves in the presence or absence of LE (Lipofundin MCT/LCT: 0.2%).

Because LE produces reactive oxygen species, a second experimental protocol was designed to investigate the involvement of superoxide anion on Lipofundin MCT/LCT-mediated attenuation of acetylcholine-induced NO-mediated relaxation in the endothelium-intact aorta [25]. After the endothelium-intact aorta was pretreated with 10^−2^ M 4,5-dihydroxy-1,3-benzenedisulfonic acid disodium salt monohydrate (tiron) for 10 min, Lipofundin MCT/LCT (0.2%) was directly added to the organ bath 20 min before phenylephrine (3 × 10^−7^ M)-induced contraction [26]. When phenylephrine-induced contraction had stabilized in the presence of tiron, incremental concentrations of acetylcholine (3 × 10^−8^ to 10^−5^ M) were added to the organ bath to generate the concentration-response curves in the presence or absence of Lipofundin MCT/LCT (0.2%).

The effects of Lipofundin MCT/LCT (0.1 or 0.2%) on the contraction induced by phenylephrine were assessed in an isolated, endothelium-intact rat aorta. Lipofundin MCT/LCT was added directly to the organ bath 15 min before the addition of phenylephrine. Incremental concentrations of phenylephrine (10^−9^ to 10^−5^ M) were added to the organ bath to generate the phenylephrine concentration-response curves in the presence or absence of Lipofundin MCT/LCT.

### 2.3. Cell Culture

Cell culture was performed as previously described [21]. Human umbilical vein endothelial cells (HUVECs; EA.hy 926 cells, American Type Culture Collection, Manassas, VA, USA) were grown in Dulbecco's modified Eagle's medium supplemented with 10% fetal bovine serum, 2 mmol/L l-glutamine, 100 IU/mL penicillin, and 10 *μ*g/mL streptomycin. Cells were cultured in 100 mm dishes and grown in a humidified 5% CO_2_ incubator. HUVECs were plated at a density of 1 × 10^7^ cells per 100 mm dish. Cells were used between passage numbers 6 and 12.

### 2.4. Cell Stimulation

Cells were plated at a density of 1 × 10^7^ cells per 100 mm dish. The cells were stimulated with acetylcholine (10^−5^ M). To detect phosphorylated endothelial NOS (eNOS), cells were treated with 10^−5^ M acetylcholine alone for 4 min or 10^−5^ M acetylcholine for 4 min after pretreatment with Lipofundin MCT/LCT (0.1 and 0.2%) for 1 hour. Then, cells were harvested and subjected to western blot analysis.

### 2.5. Western Blot Analysis

Western blot analysis was performed as previously described [21]. Briefly, cells were lysed in PROPREP protein extract solution to obtain total cell extracts. After centrifugation at 13,000 rpm for 20 min at 4°C, the protein concentration was determined by the Bradford method. Thirty micrograms of protein was subjected to 10% sodium dodecyl sulfate–polyacrylamide gel electrophoresis. The separated proteins were transferred to a polyvinylidene difluoride membrane using the SD Semi-Dry Transfer Cell System (Bio-Rad, Hercules, CA, USA). The membranes were incubated with primary antibodies (anti-eNOS and anti-phospho-eNOS antibodies; Cell Signaling Technology, Beverly, MA, USA; 4 *μ*g/mL) in 5% skim milk in TBST overnight at 4°C, and the bound antibody was detected with horseradish peroxidase-conjugated anti-rabbit IgG. The membranes were washed and then developed using the Luminol Reagent system (Animal Genetics, Suwon, Korea).

### 2.6. Materials

All drugs were of the highest purity available commercially: acetylcholine, the calcium ionophore A23187, sodium nitroprusside, l-arginine, and tiron were obtained from Sigma-Aldrich (St. Louis, MO, USA). Intralipid 20% and Lipofundin MCT/LCT 20% were donated by Fresenius Kabi Korea (Seoul, Korea) and B. Braun Korea (Seoul, Korea), respectively. All drug concentrations are expressed as the final molar concentration or as the final percentage of LE in the organ bath. The calcium ionophore A23187 was initially dissolved in dimethyl sulfoxide (final organ bath concentration: 0.05%) and subsequently diluted in distilled water. Unless stated otherwise, all other drugs were dissolved and diluted in distilled water.

### 2.7. Data Analysis

Data are expressed as the mean ± SD. Vasorelaxant responses to acetylcholine, the calcium ionophore A23187, and sodium nitroprusside are expressed as the percentage of the maximal precontraction value induced by phenylephrine. The logarithm of drug concentration (ED_50_) eliciting 50% of the maximal relaxation response was calculated with nonlinear regression analysis by fitting the concentration-response relationship for acetylcholine to a sigmoidal curve using commercially available software (Prism, version 5.0; GraphPad Software, San Diego, CA, USA). The maximum relaxant response was measured as the maximal response to each vasorelaxant. Statistical analysis for the comparison of the ED_50_ and the maximal relaxation between the control and LE-treated groups in the acetylcholine-induced relaxation was performed using the Kruskal-Wallis test followed by Dunn's multiple comparison test or the Mann-Whitney test. The effect of Lipofundin MCT/LCT on the relaxation or contraction induced by the calcium ionophore A23187, sodium nitroprusside, and phenylephrine was analyzed with a two-way repeated measures analysis of variance with Bonferroni's posttest. The effect of Lipofundin MCT/LCT alone or l-arginine plus Lipofundin MCT/LCT on the acetylcholine-induced eNOS phosphorylation in HUVECs was analyzed using one-way analysis of variance followed by Bonferroni's posttest. *N* indicates the number of rats from which descending thoracic aortic rings were obtained. *P* values less than 0.05 were considered significant.

## 3. Results

**Figure 1:**
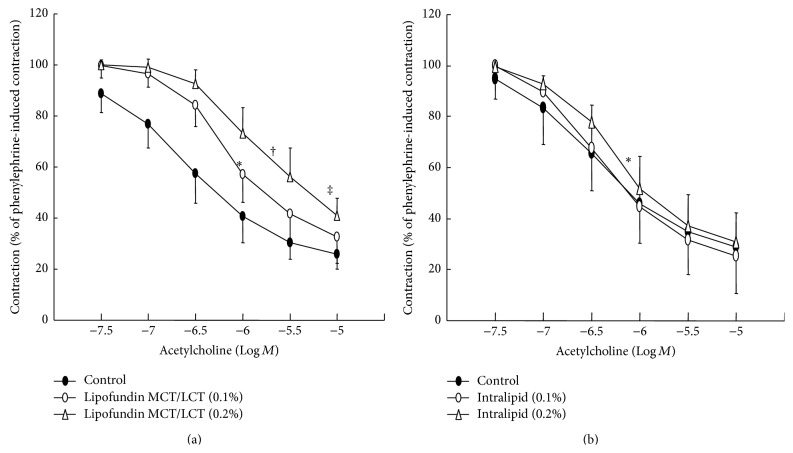
Effect of lipid emulsion (Lipofundin MCT/LCT ((a), *N* = 7) and Intralipid ((b), *N* = 7)) on the acetylcholine concentration-response curves in the endothelium-intact aorta. Data are the mean ± SD and are expressed as the percentage of maximal precontraction induced by 3 × 10^−7^ M phenylephrine. *N* indicates the number of rats from which descending thoracic aortic rings were derived. ED_50_: ^*∗*^ *P* < 0.05 and ^†^ *P* < 0.01 versus control. Maximal relaxation: ^‡^ *P* < 0.01 versus control.

**Figure 2:**
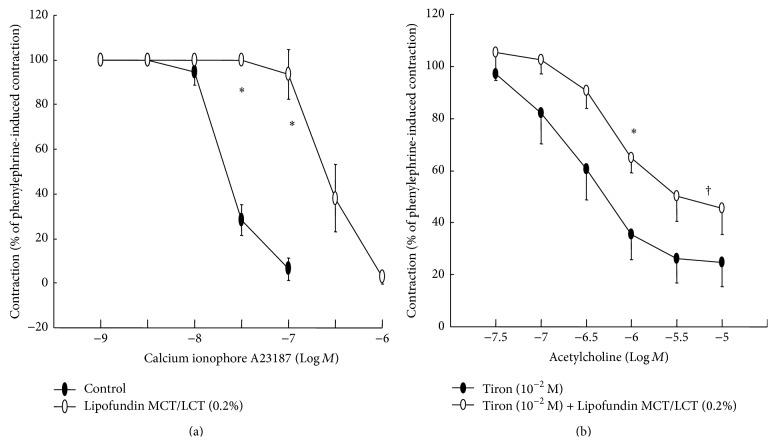
(a) Effect of Lipofundin MCT/LCT (0.2%, *N* = 6) on the calcium ionophore A23187 concentration-response curves in the endothelium-intact aorta. Data are the mean ± SD and are expressed as the percentage of maximal precontraction induced by 3 × 10^−7^ M phenylephrine. ^*∗*^ *P* < 0.001 versus control. (b) Effect of Lipofundin MCT/LCT (0.2%, *N* = 6) on acetylcholine concentration-response curves in endothelium-intact aorta pretreated with 10^−2^ M tiron. Data are the mean ± SD and are expressed as the percentage of maximal precontraction induced by 3 × 10^−7^ M phenylephrine. ED_50_: ^*∗*^ *P* < 0.01 versus tiron (10^−2^ M) alone. Maximal relaxation: ^†^ *P* < 0.01 versus tiron (10^−2^ M) alone.

**Figure 3:**
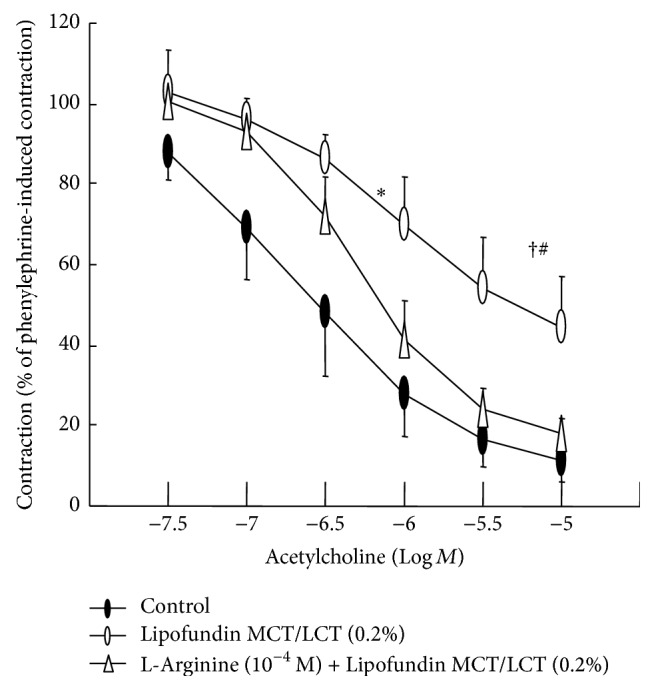
Effect of Lipofundin MCT/LCT (0.2%) alone and combined treatment with l-arginine (10^−4^ M) and Lipofundin MCT/LCT (0.2%) on the acetylcholine-induced relaxation in the endothelium-intact aorta. Data (*N* = 8) are the mean ± SD and are expressed as the percentage of maximal precontraction induced by 3 × 10^−7^ M phenylephrine. *N* indicates the number of descending thoracic aortic rings. ED_50_: ^*∗*^ *P* < 0.001 versus control. Maximal relaxation: ^†^ *P* < 0.001 versus control; ^#^ *P* < 0.05 versus l-arginine (10^−4^ M) plus Lipofundin MCT/LCT (0.2%).

**Figure 4:**
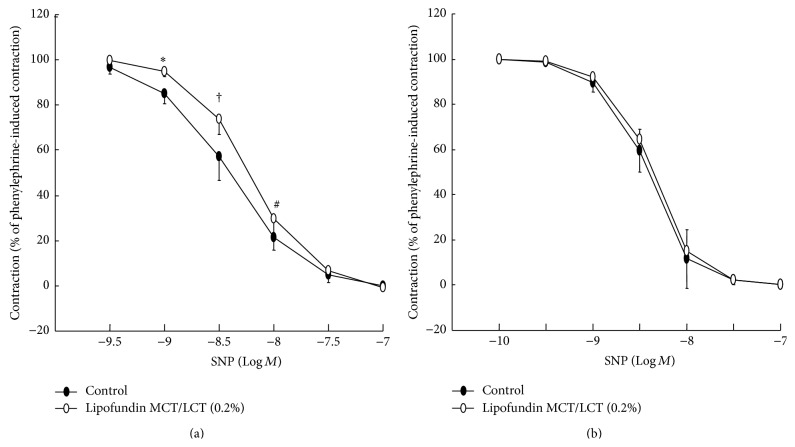
(a) Effect of Lipofundin MCT/LCT (0.2%, *N* = 6) on sodium nitroprusside (SNP) concentration-response curves in the endothelium-intact aorta. Data are the mean ± SD and are expressed as the percentage of maximal precontraction induced by 3 × 10^−7^ M phenylephrine. *N* indicates the number of rats from which descending thoracic aortic rings were derived. ^*∗*^ *P* < 0.01, ^†^ *P* < 0.001, and ^#^ *P* < 0.05 versus control. (b) Effect of Lipofundin MCT/LCT (0.2%, *N* = 6) on the SNP concentration-response curves in the endothelium-denuded aorta. Data are the mean ± SD and are expressed as the percentage of maximal precontraction induced by 10^−7^ M phenylephrine. *N* indicates the number of rats from which descending thoracic aortic rings were derived.

Lipofundin MCT/LCT (0.1% and 0.2%) attenuated acetylcholine-induced relaxation in a concentration-dependent manner (ED_50_: *P* < 0.05 versus control; Figure 1(a)), whereas 0.2% Intralipid only significantly inhibited acetylcholine-induced relaxation (ED_50_: *P* < 0.05 versus control; Figure 1(b)). In addition, Lipofundin MCT/LCT (0.2%) attenuated acetylcholine-induced maximal relaxation (*P* < 0.01 versus control; Figure 1(a)). Lipofundin MCT/LCT (0.2%) attenuated the calcium ionophore A23187-induced relaxation (*P* < 0.001 versus control; Figure 2(a)). Lipofundin MCT/LCT (0.2%) attenuated acetylcholine-induced relaxation in the endothelium-intact aorta pretreated with 10^−2^ M tiron (ED_50_ and maximal relaxation: *P* < 0.01 versus 10^−2^ M tiron alone; Figure 2(b)). Pretreatment with l-arginine (10^−4^ M) and Lipofundin MCT/LCT (0.2%) increased acetylcholine-induced maximal relaxation compared with Lipofundin MCT/LCT (0.2%) alone (*P* < 0.05; Figure 3). Lipofundin MCT/LCT (0.2%) attenuated sodium nitroprusside-induced relaxation in the endothelium-intact aorta (*P* < 0.05 versus control; Figure 4(a)), whereas Lipofundin MCT/LCT (0.2%) had no effect on sodium nitroprusside-induced relaxation in endothelium-denuded aorta (Figure 4(b)).

**Figure 5:**
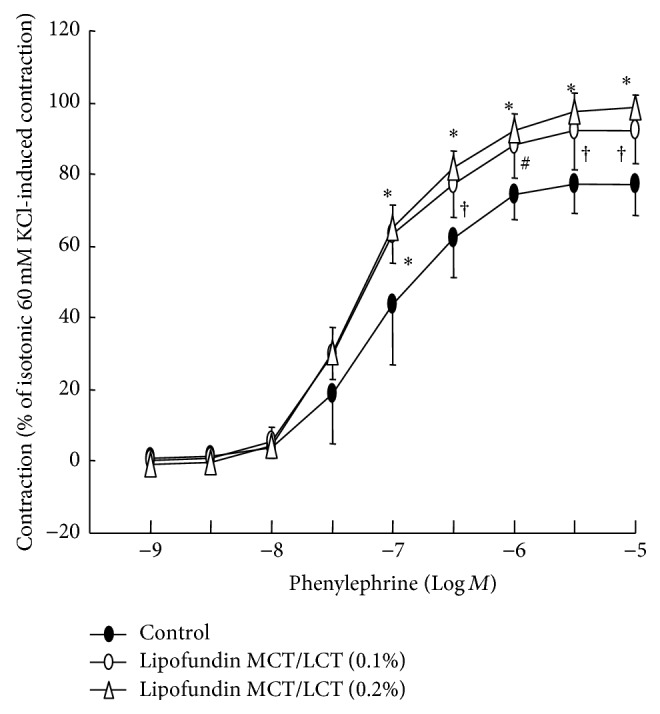
Effect of Lipofundin MCT/LCT (0.1 or 0.2%) on the phenylephrine dose-response curves in isolated endothelium-intact aorta. Data (*N* = 8) are the mean ± SD and are expressed as the percentage of maximal precontraction induced by isotonic 60 mM KCl. *N* indicates the number of descending thoracic aortic rings. ^*∗*^ *P* < 0.001, ^†^ *P* < 0.01, and ^#^ *P* < 0.05 versus control.

Lipofundin MCT/LCT (0.1 and 0.2%) increased phenylephrine-induced maximal contraction in isolated endothelium-intact aorta (*P* < 0.01 versus control; Figure 5).

**Figure 6:**
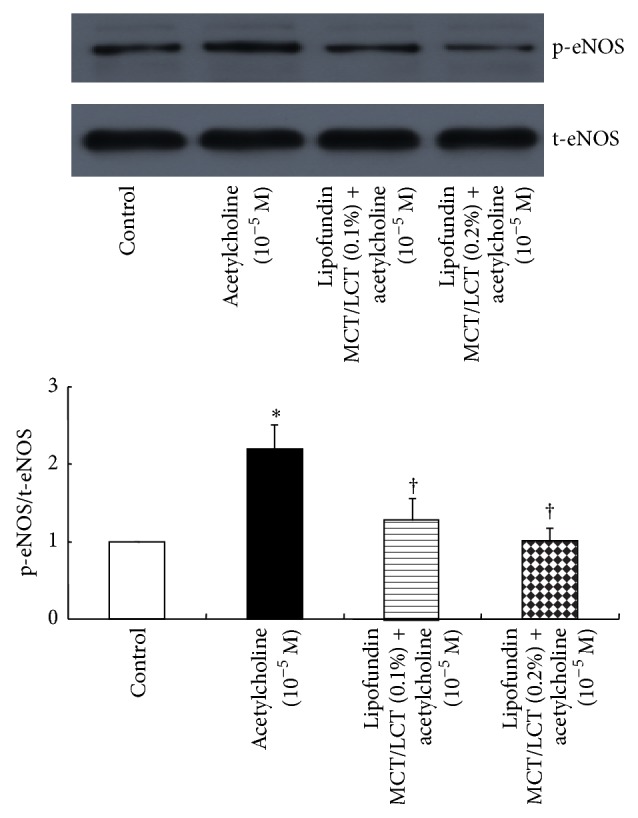
Effect of Lipofundin MCT/LCT (0.1 and 0.2%, *N* = 6) on the phosphorylation of endothelial nitric oxide synthase (eNOS) in human umbilical vein endothelial cells (HUVECs). HUVECs were treated with 10^−5^ M acetylcholine alone for 4 min or 10^−5^ M acetylcholine for 4 min after pretreatment with Lipofundin MCT/LCT (0.1 and 0.2%) for 1 hour. Data are the mean ± SD. *N* indicates the number of independent experiments. ^*∗*^ *P* < 0.001 versus control. ^†^ *P* < 0.001 versus 10^−5^ M acetylcholine alone. t-eNOS: total eNOS; p-eNOS: phosphorylated eNOS.

**Figure 7:**
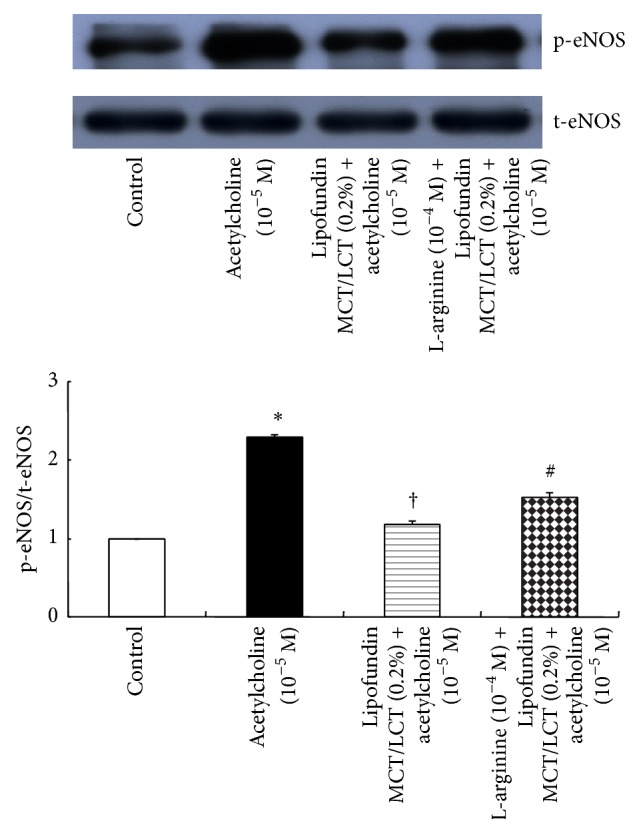
Effect of Lipofundin MCT/LCT (0.2%) alone and combined treatment with l-arginine (10^−4^ M) and Lipofundin MCT/LCT (0.2%) on the phosphorylation of endothelial nitric oxide synthase (eNOS) in human umbilical vein endothelial cells (HUVECs). HUVECs were treated with 10^−5^ M acetylcholine alone for 4 min, 10^−5^ M acetylcholine for 4 min after pretreatment with Lipofundin MCT/LCT (0.2%) for 1 hour, or 10^−5^ M acetylcholine for 4 min after treatment with Lipofundin MCT/LCT (0.2%) for 1 hour following pretreatment with 10^−4^ M l-arginine for 10 min. Data (*N* = 5) are the mean ± SD. *N* indicates the number of independent experiments. ^*∗*^ *P* < 0.001 versus control. ^†^ *P* < 0.001 versus 10^−5^ M acetylcholine alone. ^#^ *P* < 0.001 versus Lipofundin MCT/LCT (0.2%) plus acetylcholine (10^−4^ M). t-eNOS: total eNOS; p-eNOS: phosphorylated eNOS.

Acetylcholine (10^−5^ M) induced eNOS phosphorylation in HUVECs (*P* < 0.001 versus control; Figure 6), whereas Lipofundin MCT/LCT (0.1 and 0.2%) attenuated acetylcholine-induced eNOS phosphorylation (*P* < 0.001 versus 10^−5^ M acetylcholine alone; Figure 6). In addition, pretreatment with l-arginine (10^−4^ M) attenuated Lipofundin MCT/LCT (0.2%)-mediated inhibition of acetylcholine-induced eNOS phosphorylation (*P* < 0.001 versus Lipofundin MCT/LCT plus acetylcholine; Figure 7).

## 4. Discussion

This study suggests that the magnitude of LE-mediated inhibition of acetylcholine-induced NO-mediated relaxation in the isolated rat aorta appears to be dependent on the fatty acid components (long-chain and medium-chain fatty acids) of LE. The major findings of this* in vitro* study were as follows. (1) The magnitude of Lipofundin MCT/LCT-mediated inhibition of acetylcholine-induced relaxation was higher than that with Intralipid. (2) Lipofundin MCT/LCT (0.2%) attenuated the calcium ionophore A23187-induced relaxation in the endothelium-intact aorta but had no effect on sodium nitroprusside-induced relaxation in the endothelium-denuded aorta. (3) l-Arginine attenuated Lipofundin MCT/LCT-mediated inhibition of acetylcholine-induced eNOS phosphorylation in HUVECs.

Triacylglycerol, omega-3 fatty acids, and triglycerides inhibit NO production in hepatocytes, macrophages, and HUVECs, respectively [8, 10, 11]. The formulation of fatty acids in Intralipid 20% includes 100% long-chain fatty acids (53% linoleic acid, 24% oleic acid, 11% palmitic acid, 4% stearic acid, and 8% alpha-linolenic acid), whereas that of Lipofundin MCT/LCT 20% contains 50% medium-chain fatty acids (30% caprylic acid and 20% capric acid) and 50% long-chain fatty acids (26.5% linoleic acid, 11.9% oleic acid, 6.1% palmitic acid, 3.5% alpha-linolenic acid, and 2.2% stearic acid) [27, 28]. Considering previous reports, in our current study, Lipofundin MCT/LCT-mediated enhanced inhibition of acetylcholine-induced NO-mediated relaxation may be associated with differences in fatty acid components that are contained in LE such as increased medium-chain fatty acids and decreased long-chain fatty acids [8, 10, 11, 17, 20, 27, 28]. Further studies are required to determine which fatty acid component of Lipofundin MCT/LCT is mainly responsible for the Lipofundin MCT/LCT-mediated attenuation of acetylcholine-induced NO-mediated relaxation. Intralipid (0.2 and 1%, 2 mg/kg) and free fatty acids attenuate acetylcholine-induced relaxation in isolated rat femoral artery, whereas free fatty acids have no effect on NO donor sodium nitroprusside-induced relaxation [9, 17]. Triglyceride emulsion inhibits endothelin-3-induced NO synthesis in HUVECs by inhibiting the increase in cytosolic intracellular calcium [8]. Increased intracellular calcium levels are required for NOS activation for production of NO [29]. The calcium ionophore A23187 enhances free intracellular calcium levels and therefore can activate NOS, leading to NO formation [29]. The NO donor sodium nitroprusside activates guanylyl cyclase, leading to relaxation [29]. Lipofundin MCT/LCT slightly attenuated sodium nitroprusside-induced relaxation in the endothelium-intact aorta, suggesting that Lipofundin MCT/LCT may interfere with the transfer of NO from the endothelium to vascular smooth muscle. Similar to previous reports, Lipofundin MCT/LCT inhibited relaxation induced by the endothelium-dependent vasodilators acetylcholine and the calcium ionophore A23187 but had no effect on the endothelium-independent vasodilator sodium nitroprusside in the endothelium-denuded aorta, suggesting that Lipofundin MCT/LCT inhibits a site upstream of guanylyl cyclase activation of vascular smooth muscle [17, 18].

SMOFlipid, which includes 61% long-chain triglycerides and 39% medium-chain triglycerides, decreases the phosphorylation of eNOS that is induced by levobupivacaine, and free fatty acids including palmitic acid and oleic acid decrease eNOS activity [20, 30, 31]. Similar to a previous report, Lipofundin MCT/LCT, which includes medium-chain triglycerides, attenuated eNOS phosphorylation induced by acetylcholine in HUVECs in our current study [20]. l-Arginine is a competitive inhibitor of NOS inhibitors such as L-NAME [32]. In the present study, l-arginine inhibited Lipofundin MCT/LCT-mediated attenuation of acetylcholine-induced eNOS phosphorylation, suggesting that this l-arginine-mediated partial reversal of eNOS phosphorylation may be associated with the reversal of inhibition of eNOS induced by Lipofundin MCT/LCT in HUVECs. However, as we used both rat aorta for tension measurement and HUVECs for detection of eNOS phosphorylation in the current study, tissue heterogeneity should be considered when interpreting our current results. Consistent with results obtained from western blotting using l-arginine, which significantly decreased Lipofundin MCT/LCT (0.2%)-induced attenuation of acetylcholine-induced relaxation in our current study, Lipofundin MCT/LCT-mediated inhibition of acetylcholine-induced relaxation appears to be partially associated with inhibition of eNOS [29, 32]. In addition, the Lipofundin MCT/LCT-mediated enhancement of phenylephrine-induced contraction in isolated endothelium-intact aorta observed in the current study may be partially due to Lipofundin MCT/LCT-mediated inhibition of endothelial NO.

In previous* in vitro* studies, the antioxidant vitamin C partly restores the impaired acetylcholine-induced relaxation induced by free fatty acids, and triacylglycerol attenuates NO production by increasing reactive oxygen species production [10, 17]. Lipofundin MCT/LCT (0.5 and 0.95%)-induced dichlorofluorescein production in HUVECs is higher than Intralipid-induced dichlorofluorescein production [25]. However, in our current study, the low molecular-weight superoxide anion scavenger tiron did not abolish Lipofundin MCT/LCT-mediated attenuation of acetylcholine-induced relaxation, suggesting that Lipofundin MCT/LCT (0.2%)-mediated attenuation of acetylcholine-induced NO-mediated relaxation may not be associated with superoxide anion production. The different results between the current study and previous studies may be due to differences in the LE concentration (0.2% versus 0.5 or 0.95%) and the reactive oxygen species inhibitor (vitamin E analogue tiron versus vitamin C) [17, 25].

The clinical implications of Lipofundin MCT/LCT-induced enhanced attenuation of endothelium-dependent NO-mediated relaxation should be cautiously interpreted because the aorta, which is considered a conduit vessel, was used in this* in vitro* experiment, whereas small-resistance arterioles regulate organ blood flow. The proposed mechanisms associated with triglyceride microemulsion-mediated reversal of bupivacaine toxicity include LE-mediated sequestration (lipid sink), cardiotonic effects, supply of fatty acids, and drug redistribution [33–35]. These factors may have impacted our current results. However, considering previous reports, because local anesthetics such as levobupivacaine, ropivacaine, and mepivacaine induce NO release, our results suggest that, compared with Intralipid, Lipofundin MCT/LCT may have more favorable effects regarding LE-mediated vascular tone recovery from vascular collapse (or severe hypotension) induced by NO produced by a toxic dose of a local anesthetic [19, 21, 22]. The enhanced Lipofundin MCT/LCT-mediated attenuation of endothelial NO-induced relaxation observed in the current study may contribute to the increased blood pressure, systemic vascular resistance, and enhanced left ventricular systolic pressure produced by intravenous administration of Lipofundin MCT/LCT compared with Intralipid that was observed in previous* in vivo* studies [12, 18].

In conclusion, Lipofundin MCT/LCT attenuated acetylcholine-induced NO-mediated relaxation via an inhibitory effect on the endothelium including eNOS that is upstream of guanylyl cyclase activation, and this seems to be associated with the fatty acid components (decreased long-chain fatty acids and increased medium-chain fatty acids) contained in Lipofundin MCT/LCT.
